# Exploring the biomechanical mechanisms of neuromuscular joint facilitation intervention in patients with knee osteoarthritis: a finite element analysis

**DOI:** 10.3389/fresc.2026.1945210

**Published:** 2026-09-29

**Authors:** Yu Qu, Hualong Xie, Peipei Wang, Xin Zhang, Shan Liu, Ming Huo

**Affiliations:** 1 Qilu Hospital of Shandong University, Jinan, China; 2 School of Rehabilitation Science and Engineering, University of Health and Rehabilitation Sciences, Qingdao, Shandong, China; 3 Wangjing Hospital, China Academy of Chinese Medical Sciences, Beijing, China; 4 Department of Rehabilitation Medicine, Qingdao Central Hospital, University of Health and Rehabilitation Sciences (Qingdao Central Medical Group), Qingdao, China; 5 Beijing Chaoyang District Sanhuan Cancer Hospital, Beijing, China; 6 Shandong Key Laboratory of Neurorehabilitation, University of Health and Rehabilitation Sciences, Qingdao, China; 7 Jilin Province Power Hospital, Changchun, China; 8 Xi'an Electric Power Central Hospital, Xi'an City, China

**Keywords:** biomechanics, finite element analysis, knee osteoarthritis, neuromuscular joint facilitation, resistance training

## Abstract

**Introduction:**

Knee osteoarthritis (KOA) causes joint pain and gait disorders in older adults. Exercise is an effective non-pharmaceutical therapy. Neuromuscular joint facilitation (NJF) improves knee joint function by combining neuromuscular facilitation with intra-articular joint motion; however, its biomechanical mechanism remains unclear. Finite element analysis, the mechanisms by which two NJF movement patterns—knee extension with lateral tibial rotation and knee flexion with medial tibial rotation—affect the stress distribution on the meniscus and major ligaments in patients with KOA.

**Methods:**

Six KOA patients were recruited. Inertial motion capture and handheld dynamometer measured tibial rotation angles and force conditions: active knee extension/flexion (AKE/AKF) pattern, distally single-resisted knee extension and flexion (DKE/DKF) pattern, and NJF technique (double-resisted movement). A knee finite-element model was built from one subject's CT/MRI. Loaded parameters were used to analyze the stress distribution characteristics of the medial collateral ligament (MCL), lateral collateral ligament (LCL), anterior/posterior cruciate ligament (ACL/PCL), and medial/lateral menisci at flexion angles of 0°, 30°, 60°, and 90°.

**Results:**

Knee rotation ICCs from inertial motion capture were all >0.75 (*P* < 0.05). In the knee flexion-medial tibial rotation pattern, at all flexion angles, meniscal stress was highest during AKF and lowest during NJF; conversely, ligament stress was lowest during AKF and highest during NJF. In the extension-lateral tibial rotation mode, meniscal stress was highest during AKE and lowest during NJF, whereas ligament stress changes were angle-dependent. The NJF technique can produce a traction effect along the long tibial axis by coordinating proximal and distal tibial resistances. This applies a force of approximately 30° to the long axis of the tibia, thereby reducing the load within the joint capsule.

**Discussion:**

The NJF technique can redistribute load between the meniscus and ligaments in this subject-specific finite element model, reducing peak meniscal load while moderately increasing ligament mechanical stimulation. These computational findings suggest a distinctive biomechanical profile of NJF compared with traditional resistance training in this subject-specific model. This provides a preliminary theoretical basis for future research, but the clinical relevance of these biomechanical observations requires confirmation through prospective clinical trials.

## 1 Introduction

Knee osteoarthritis (KOA) is a degenerative disease affecting the articular cartilage and subchondral bone. It is frequently accompanied by secondary bone hyperplasia. This leads to joint pain, swelling, deformity, dysfunction, and gait abnormalities (1). The etiology of KOA is unknown; however, it is associated with age, obesity, trauma, and genetics (2). In recent years, with an increase in population age and obesity rates, the number of patients with KOA has gradually increased (3). KOA results in disability in nearly 365 million individuals worldwide, and by 2050, cases are projected to surge by 74.9% (95% confidence interval [CI]: 59.4–89.9) (GBD 2021 Osteoarthritis Collaborators, 2023). By the end of 2023, the proportion of people aged 60 years and above with KOA in China will reach 21.1% (4). The clinical characteristics of KOA primarily include pain and gait disorders. These symptoms gradually worsen with disease progression. They severely affect the quality of life and being a major cause of disability (5).

Exercise therapy for KOA is widely used worldwide, and various guidelines and evidence-based medicines recommend it to relieve pain and improve joint function. Joint movement training, proprioception and balance training, and gait training can improve joint function, reduce disability rates, delay articular cartilage degeneration, and postpone the need for surgical intervention in patients with KOA (2, 6).

Neuromuscular joint facilitation (NJF) is a novel exercise therapy based on kinesiology that combines the facilitatory elements of proprioceptive neuromuscular facilitation (PNF) with those of intra-articular accessory motion. It improves joint function through passive, active, and resistant movements. NJF not only facilitates nerves and muscles but also improves intra-articular motion. Previous studies on NJF have primarily focused on clinical and functional outcomes, such as pain relief, muscle strength improvement, and gait enhancement (7, 8). While these findings support the therapeutic efficacy of NJF, they are inherently limited in elucidating the underlying biomechanical mechanisms. Specifically, it remains unclear how the distinctive double-resistance pattern of NJF modulates internal stress distribution across intra-articular structures, such as the menisci and ligaments during movement.

Several critical questions remain unresolved: (1) whether the therapeutic benefits of NJF arise from muscle activation, joint traction, or altered load transmission pathways; (2) how NJF differs quantitatively from conventional single-resisted or non-resisted movements in terms of intra-articular stress patterns; and (3) how these effects vary across knee flexion angles.

Computational biomechanics provides a powerful methodology for obtaining information that is difficult to acquire through experimental approaches alone (9). *In vivo* experiments are often prohibitively costly, time-consuming and technically complicated (10). In this context, finite element modeling (FE) offers a unique, non-invasive, and validated tool. It can analyze tissue loading, estimate stress and strain distributions, and predict regions at increased risk of mechanical failure due to OA progression in a subject-specific way (11). Subject-specific geometries can be obtained through magnetic resonance (MR) imaging and discretized into FE. Subsequently, appropriate material models and properties are assigned to the individual knee components, with adequate initial and boundary conditions and interactions between components being imposed. Through FE simulations, researchers can test hypotheses and elucidate cause-effect relationships between various loading conditions and the resulting soft tissue responses (12). This computational approach allows us to isolate the specific mechanical effects of NJF's distinctive double-resistance pattern and to systematically compare it with active and single-resisted movements.

Therefore, this study aimed to investigate stress changes and mechanisms of action in the major ligaments, menisci, and articular surfaces during NJF knee extension-lateral tibial rotation and knee flexion-medial tibial rotation patterns using finite element analysis.

## 2 Materials and methods

### 2.1 Study subjects

Six patients with KOA were recruited for this study with the following characteristics(two males and four females). Their mean age: 69.2 ± 4.3 years, mean height: 162.8 ± 4.2 cm, mean weight: 70.6 ± 12.4 kg. The mean disease duration was 5 ± 2.5 years (range: 2–8 years). Unilateral knee symptoms were reported(4 right, 2 left). According to the Kellgren-Lawrence grade scale, four parients were classified as grade I and two as grade II. The mean VAS pain score during weight-bearing of 4 ± 1.9(range: 2–7). No changes in medication regimen and no intra-articular injections within 6 months prior to the study.

All enrolled patients met the inclusion criteria and signed informed consent forms. This study was approved by the Ethics Committee of the University of Health and Rehabilitation Sciences, Qingdao Municipal Hospital (No. 2024-KY-002), and was registered with the Chinese Clinical Trial Registry (Registration No.: ChiCTR2400086711).

The inclusion criteria were based on the 2018 diagnostic criteria for KOA: (1) recurrent knee pain within the past month; (2) radiographic findings showing joint space narrowing, subchondral bone sclerosis and/or cystic changes, and marginal osteophyte formation; (3) age ≥50 years; (4) morning stiffness <30 min; and (5) crepitus during activity. KOA was diagnosed if criteria (1) and (2), (1), (4), and (5), or (1), (3), (4), and (5) were met. Additionally, according to the Kellgren–Lawrence grading scale, patients with stage I or II anteroposterior and lateral knee radiographs were included.

The exclusion criteria for patients with KOA were: (1) failure to meet diagnostic and inclusion criteria; (2) concomitant knee sprain or other trauma; (3) severe knee varus or valgus, or conditions such as rheumatoid arthritis and gout; (4) foot and ankle deformities, pain, or other lesions affecting normal gait; (5) severe visceral diseases, nervous system diseases, other serious metabolic disorders, or bone tumors; (6) treatment with immunosuppressants or local/systemic corticosteroids within 3 weeks before the study; (7) psychiatric disorders or intellectual disability; and (8) Kellgren–Lawrence grade >II.

### 2.2 Experimental methods

First, the reliability of the kinematic parameters measured using a wireless inertial motion capture system under the three intervention methods listed below was analyzed. Second, kinematic parameters under the three interventions were collected from all patients using a wireless inertial motion capture system, and the mechanical parameters during the interventions were collected using a handheld dynamometer. Finally, we established a finite element model. The kinematic and mechanical parameters were input into the model. This allowed us to analyze the stress changes and mechanisms of action on the major ligaments, menisci, and articular surfaces under different intervention methods (Figure 1).

**Figure 1:**
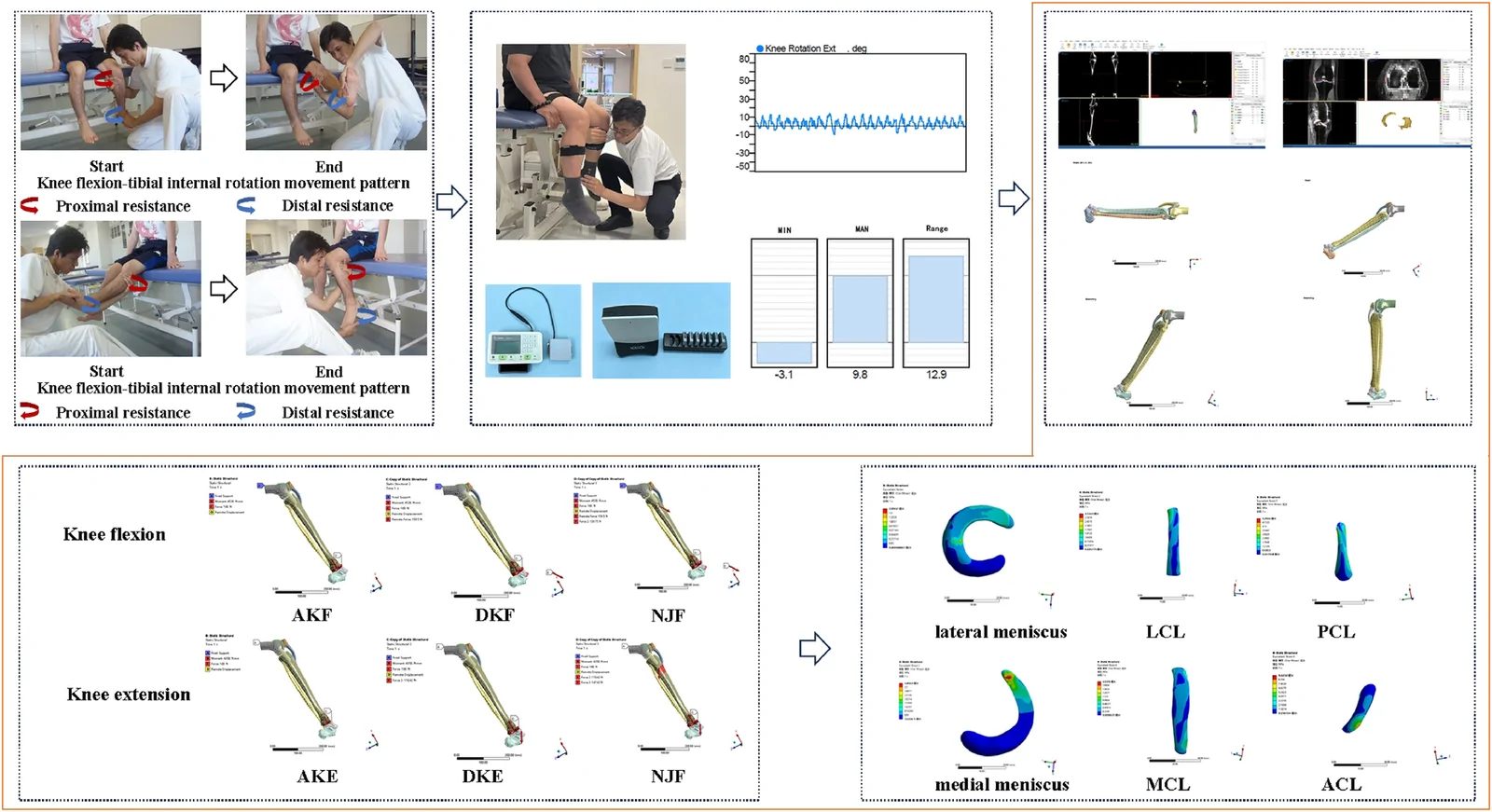
Finite element modeling workflow. AKE, active knee extension; AKF, active knee flexion; DKE, distally single-resisted knee extension; DKF, distally single-resisted knee flexion; NJF technique, neuromuscular joint facilitation; LCL, lateral collateral ligament; MCL, medial collateral ligament; ACL, anterior cruciate ligament; PCL, posterior cruciate ligament.

### 2.3 Model validation

This study validated the effectiveness of the finite element model by systematically comparing its simulation results with data from previous studies. The validation protocol was based on previous study designs. At 0° of flexion, a 134 N posterior load was applied to the femur to simulate the anterior drawer test of the knee joint and clinically common loading conditions, and to facilitate comparative validation with other similar studies.

### 2.4 Mesh convergence testing

To determine a reasonable mesh size for the finite element model, simulation analyses were conducted based on the baseline model. Since mesh size directly affects the accuracy of finite element calculations and the reliability of the results, mesh convergence verification was prioritized on the basis of the experimental model. Specifically, while keeping the model geometry, material parameters, boundary conditions, and loading conditions consistent, the same model was discretized and calculated using different mesh sizes, so as to exclude interference from other factors and examine only the effects of mesh size variation on stress and displacement results. By extracting stress and displacement data under different mesh schemes and conducting comparative analysis, the convergence and credibility of the mesh were evaluated.

### 2.5 Intervention methods

#### 2.5.1 Active knee extension/flexion (AKE/AKF) pattern

Patients were seated with their knees flexed at 90°. Two movement patterns were observed in the absence of resistance. For the knee extension-lateral tibial rotation pattern, the knee started at 90° flexion and extended, while the tibia was externally rotated until full extension. For the knee flexion-medial tibial rotation pattern, the knee was fully extended and flexed, while the tibia was internally rotated until 90° flexion.

#### 2.5.2 Distally single-resisted knee extension and flexion (DKE/DKF) pattern

Patients were seated with their knees flexed at 90°. For the knee extension-lateral tibial rotation pattern, the knee started at 90° flexion and extended, while the tibia was externally rotated until full extension. For the knee flexion-medial tibial rotation pattern, the knee was fully extended and flexed, while the tibia was internally rotated until 90° flexion. During movement, resistance was applied to the distal tibia at a 30° angle to the tibial long axis in the direction opposite to the movement.

#### 2.5.3 NJF technique (double-resistance movement)

Patients were seated with their knees flexed at 90°. For the extension-lateral tibial rotation pattern, the knee started at 90° flexion and extended, while the tibia was externally rotated until full extension. Resistance was simultaneously applied to the proximal and distal tibias. Proximal resistance was applied on the medial side below the tibial plateau at an angle of 30° to the tibial long axis in the direction opposite to the movement. The locations and directions of the distal resistance were identical to those of the DKE pattern. For the knee flexion-medial tibial rotation pattern, the knee was fully extended and flexed, while the tibia was internally rotated until 90° flexion. Resistance was applied to both the proximal and distal tibiae. Proximal resistance was applied to the medial aspect of the posterior tibia at a 30° angle to the tibial long axis in the direction opposite to the movement. Distal resistance was applied as in the DKF pattern.

A flowchart of the study is shown in Figure 1.

### 2.6 Outcome measures

#### 2.6.1 Quantification of tibial kinematic and mechanical parameters

A wireless inertial motion capture system (Ultium Motion, USA) was placed on the sacral region, bilateral midthigh, bilateral lower leg (two finger widths below the fibular head), and bilateral back of the feet (just below the ankle). For calibration, the patient was seated with the hip and knee flexed at 90°. The total rotation angle of the tibia was measured during AKE/AKF, DKE/DKF, and NJF. A handheld dynamometer (ANIMA, Japan) was used to measure the force at the corresponding locations during the DKE/DKF and NJF interventions. All interventions were performed by the same physiotherapist with over 5 years of NJF experience.

#### 2.6.2 Biomechanical changes of the knee joint

A finite element model was established and loaded with tibial kinematic and mechanical parameters to evaluate stress changes in the meniscus, medial collateral ligament (MCL), lateral collateral ligament (LCL), anterior cruciate ligament (ACL), and posterior cruciate ligament (PCL).

The computed tomography (CT) and magnetic resonance imaging (MRI) data from one subject were used to build the finite element model. DICOM-format raw data were imported into Mimics 21.0 (Materialise, Belgium) to create a 3D mesh model. Each part was exported as an STL file and imported into Geomagic 2017 (Geomagic, USA) for surface treatment and model optimization. Subsequently, they were imported into SolidWorks 2021 (Dassault Systèmes) for assembly. Finally, they were imported into ANSYS 21.0 (Ansys, USA). The material properties were assigned based on the literature (Table 1) (13, 14). A mesh was generated to create a valid analysis file. The tibial kinematic and mechanical parameters from the three interventions were used to evaluate the stress changes in the meniscus, MCL, LCL, ACL, and PCL.

**Table 1:** Material properties .

Components	Young's modulus E (MPa)	Poisson's ratio (*ν*)
Cortical bone	12,000	0.3
Cartilage	5	0.46
Meniscus	59	0.49
Anterior cruciate ligament	116	0.3
Posterior cruciate ligament	87	0.3
Medial collateral ligament	48	0.3
Lateral collateral ligament	48	0.3
Patellar ligament	87	0.3

It is important to emphasize that this finite element model was constructed from imaging data of a single subject, and thus the stress distributions presented in this study are subject-specific rather than population-based.

#### 2.6.3 Loading conditions

For each of the three interventions and two movement patterns, stress changes were observed at 0°, 30°, 60°, and 90° flexion. The proximal femur was fixed in all models. A force of 168 N was applied along the long tibial axis at the distal tibial joint to simulate muscle compression (ISO, 2014).

All soft-tissue components—including the menisci, articular cartilage, and ligaments—were modeled as linear elastic, isotropic, and homogeneous materials. This simplification was adopted because the primary analytical objective was to compare the relative stress distributions among the three intervention conditions within the same subject-specific geometry, rather than to predict absolute tissue failure. Under this framework, the uniform application of linear properties across all simulated conditions allows valid relative comparisons while maintaining computational efficiency and consistency with previously validated knee FE models (11, 13, 14). The 30° loading angle was derived directly from the established NJF clinical protocol (8), as this angle optimally combines axial traction with rotational torque to facilitate intra-articular motion. Importantly, the force magnitudes applied in the FE model were not arbitrarily selected but were measured experimentally using a handheld dynamometer during the motion capture sessions (see Section 3.2 for force values). These subject-specific force inputs enhance the clinical relevance of the computational simulations. The proximal femur was fixed in all models, and a vertical displacement constraint was applied at the distal talus to simulate seated weight-bearing conditions and to neutralize vertical forces from resistance, in accordance with ISO 14243-3:2014.

Active muscle forces were not incorporated into the model to isolate the mechanical effects of the three resistance modalities under controlled external loading. While muscles—particularly quadriceps, hamstrings, and gastrocnemius—modulate joint contact mechanics *in vivo* (15), our primary objective was to compare relative stress distributions attributable to the resistance patterns themselves, rather than to simulate full physiological activation. This approach is consistent with previous comparative FE studies (13, 14).

#### 2.6.4 AKE/AKF

No external forces were applied. Torque was applied to the distal tibial joint region to simulate the tibial torsion angle during motion. Meanwhile, a remote displacement constraint was applied at the talus to restrict vertical displacement of the knee joint, thus neutralizing vertical forces from both single and double resistances.

## 3 Results

Table 1 summarizes the material properties (Young's modulus and Poisson's ratio) of the cortical bone, cartilage, meniscus, knee ligaments (ACL, PCL, MCL, and LCL), and the patellar ligament used in the NJF knee joint model.

### 3.1 Results of model validation

Under a 134 N posterior load applied to the femur, the displacements of the knee joint model in the anterior-posterior (AP), proximal-distal (PD), and medial-lateral (ML) directions were 3.7919 mm, 0.28066 mm, and 1.0244 mm, respectively. The varus-valgus (VV) and external-internal (EI) angles were 0.37398° and 3.3479°, respectively. The maximum stresses in the ACL, PCL, MCL, and LCL were 24.111 MPa, 6.4075 MPa, 0.48165 MPa, and 5.5544 MPa, respectively.

Song et al. (16) validated the force and stress distribution of an ACL finite element model based on cadaveric experimental data. Li et al. used a 134 N load for anterior drawer test validation and obtained a maximum posterior tibial displacement of 4.9129 mm and a maximum ACL stress of 19.253 MPa (17). The results of this study are consistent with the displacement response trends reported in previous studies, indicating that this model has good biomechanical predictive ability under physiological loading conditions.

### 3.2 Results of mesh convergence testing

Comparison of the calculation results under different mesh schemes showed that the displacement results remained essentially unchanged as the mesh size varied. Under the premise that displacement remained basically unchanged, when the stress variation was less than 5%, further mesh refinement could be considered to have little effect on the calculation results, indicating that the mesh scheme had good accuracy and credibility. Based on a comprehensive comparison of the calculation results under different mesh sizes, an overall mesh size of 4 mm was ultimately selected, with a mesh size of 0.5 mm applied to local critical regions. This mesh scheme has relatively high accuracy and can accurately reflect the stress and displacement characteristics of the model (Table 2).

**Table 2:** Mesh convergence analysis results.

Mesh convergence	Number of elements	Number of nodes	Equivalent stress	Displacement	Percentage change
9	40,131	83,369	29.106	6.1877	
8	46,251	98,521	27.594	6.1847	−0.05195
7	51,547	1,00,326	26.312	6.192	−0.04646
6	61,234	1,25,891	25.121	6.1921	−0.04526
5	78,756	1,48,767	24.531	6.1899	−0.02349
4	88,107	1,65,164	24.111	6.1898	−0.01712
3	1,01,520	1,82,631	23.611	6.1886	−0.02074

### 3.3 Reliability analysis

The reliability analysis of kinematic parameters obtained from the wireless inertial motion capture system demonstrated good consistency across all three movement interventions. Intraclass correlation coefficients exceeded 0.75 for all parameters (P < 0.05), indicating acceptable measurement reliability (Table 3).

**Table 3:** ICC of tibial rotation angle during different interventions (°).

Intervention	Pattern	Test 1	Test 2	ICC	95%CI	*P*-value	SEM	MDC
Non-resisted	AKE	17.63 ± 9.41	17.88 ± 8.08	0.856	(0.287, 0.979)	0.007	3.32	9.20
	AKF	17.17 ± 8.46	18.31 ± 7.29	0.849	(0.261, 0.977)	0.008	3.07	8.50
Single-resisted	DKE	17.10 ± 4.88	16.57 ± 6.30	0.836	(0.222, 0.975)	0.010	2.26	6.26
	DKF	19.75 ± 8.05	18.78 ± 6.70	0.798	(0.109, 0.969)	0.016	3.31	9.17
NJF	KE	12.35 ± 4.29	12.00 ± 2.99	0.822	(0.176, 0.973)	0.012	1.54	4.27
	KF	21.67 ± 3.48	20.30 ± 5.63	0.801	(0.117, 0.969)	0.015	2.03	5.62

AKE, active knee extension; AKF, active knee flexion; DKE, distally single-resisted knee extension; DKF, distally single-resisted knee flexion; KE, knee extension; KF, knee flexion; SEM, standard error of measurement; MDC, minimal detectable change.

In addition to ICC values, we calculated the standard error of measurement (SEM) and minimal detectable change (MDC) to evaluate absolute measurement precision. SEM was calculated as SD × √(1 -ICC), where SD represents the pooled standard deviation of the two test sessions. MDC was calculated as SEM×1.96 × √2. The SEM values for the six movement conditions ranged from 1.54° to 3.32°, and the corresponding MDC values ranged from 4.27° to 9.20° (Table 3). These indices indicate that while the measurement system demonstrated good relative reliability, the absolute precision of the tibial rotation measurements should be considered when interpreting the kinematic inputs to the finite element model.

### 3.4 Quantitative analysis of kinematic parameters and resistance

Non-resisted movements produced tibial rotation angles of 17.76° for the AKE lateral-rotation pattern and 17.74° for the AKF medial-rotation pattern. Under single-resisted conditions, the DKE lateral-rotation pattern generated a rotation angle of 16.83° with distal tibial resistance of 176.42 N, while the DKF medial-rotation pattern produced 19.27° of rotation with distal resistance of 159.50 N. During NJF, knee extension with lateral rotation produced 12.18° of tibial rotation, accompanied by proximal and distal resistances of 119.92 N and 147.42 N, respectively. Knee flexion with medial rotation produced 20.98° of rotation, with proximal and distal resistances of 118.92 N and 139.75 N.

### 3.5 Stress changes across interventions

Finite element analysis revealed distinct stress patterns across the three interventions. For both flexion-medial rotation and extension-lateral rotation modes at 0°, 30°, 60°, and 90°, stresses in the lateral and medial menisci were consistently highest during non-resisted movement and lowest during NJF. In contrast, ligament stresses (LCL, MCL, ACL, and PCL) were generally lowest during non-resisted movement and highest during NJF, although angle-specific variations were observed.

For the extension-lateral rotation mode, LCL stress was lowest during distal-resisted movement at 0° and 90°, and highest during NJF at 0°. At 30° and 60°, LCL stress peaked during non-resisted movement and was lowest during NJF. MCL stress was lowest during non-resisted movement at 0° and 30°, highest during NJF at 0°, and highest during distal-resisted movement at 60° and 90°. ACL stress was lowest during non-resisted movement at 0° and 90°, highest during NJF at 0° and 90°, and peaked during distal-resisted movement at 30°. PCL stress was lowest during non-resisted movement at 0°, 60°, and 90°, and highest during NJF at these angles. At 30°, it was lowest during non-resisted movement and highest during distal-resisted movement (Tables 4, 5).

**Table 4:** Knee joint stress in flexion-tibial internal rotation pattern (mpa).

Angle	Intervention	Lateral meniscus	Medial meniscus	LCL	MCL	PCL	ACL
0°	**AKF**	0.15733^a^	0.33006^a^	0.54451^b^	0.30965^b^	1.2666^b^	1.652^b^
	**DKF**	0.033705	0.12959	0.55328	0.35323	1.3041	1.8676
	**NJF**	0.000221^b^	0.00065^b^	0.66759^a^	0.44536^a^	1.3382^a^	2.9043^a^
30°	**AKF**	0.41185^a^	0.44712^a^	0.64818^b^	0.28877^b^	0.87581^b^	0.82412^b^
	**DKF**	0.23768	0.27903	0.65269	0.3468	1.2209	1.2426
	**NJF**	0.19442^b^	0.1166^b^	0.73639^a^	0.4^a^	1.7218^a^	2.4459^a^
60°	**AKF**	0.44335^a^	0.50659^a^	0.64807^b^	0.23834^b^	0.7097^b^	0.76608^b^
	**DKF**	0.22438	0.38124	0.68311	0.26877	0.90792	1.0924
	**NJF**	0.12119^b^	0.19721^b^	0.82425^a^	0.29794^a^	1.3108^a^	2.616^a^
90°	**AKF**	0.43756^a^	0.22774^a^	0.65205^b^	0.26643^b^	0.89928^b^	0.44764^b^
	**DKF**	0.21098	0.16472	0.7117	0.2872	1.1976	0.87352
	**NJF**	0.11755^b^	0.077428^b^	0.98078^a^	0.30408^a^	1.89^a^	2.2582^a^

All stress values presented in this table are simulation-derived outputs from the finite element model, not statistically estimated parameters or *in vivo* measurements. Values bolded the highest and lowest values.

a Values marked with an indicate the overall maximum stress.

b Values marked with indicate the lowest stress among the three intervention conditions.

AKF, active knee flexion; DKF, distally single-resisted knee flexion; NJF technique, neuromuscular joint facilitation.

**Table 5:** Knee joint stress in extension-tibial external rotation pattern (mpa).

Angle	Intervention	Lateral meniscus	Medial meniscus	LCL	MCL	PCL	ACL
0°	**AKE**	0.24177^a^	0.40388^a^	0.60501	0.28871^b^	1.3301^b^	1.2755^b^
	**DKE**	0.062779	0.18799	0.5628^b^	0.30585	1.4864	1.459
	**NJF**	0.000173^b^	0.000171^b^	0.81845^a^	0.34547^a^	3.58^a^	2.4197^a^
30°	**AKE**	0.25918^a^	0.64658^a^	0.67405^a^	0.27823^a^	2.1778^b^	1.217
	**DKE**	0.079958	0.46649	0.65441	0.26649	2.4711^a^	1.2592^a^
	**NJF**	0.002116^b^	0.13724^b^	0.54652^b^	0.18814^b^	2.4176	0.94313^b^
60°	**AKE**	0.1592^a^	0.67599^a^	0.7024^a^	0.22829	1.6939^b^	1.0948^a^
	**DKE**	0.062803	0.46599	0.63734	0.2534^a^	1.974	0.87368^b^
	**NJF**	0.002952^b^	0.021252^b^	0.56956^b^	0.20423^b^	2.4496^a^	0.91689
90°	**AKE**	0.12877^a^	0.69025^a^	0.71298^a^	0.23596^b^	1.578^b^	1.0419
	**DKE**	0.037555	0.39771	0.63353^b^	0.28596^a^	1.8923	0.80504^b^
	**NJF**	0.000376^b^	0.000569^b^	0.64642	0.26123	2.9544^a^	1.2309^a^

All stress values presented in this table are simulation-derived outputs from the finite element model, not statistically estimated parameters or *in vivo* measurements. Values bolded the highest and lowest values.

a Values marked with an indicate the overall maximum stress.

b Values marked with indicate the lowest stress among the three intervention conditions.

AKE, active knee extension; DKE, distally single-resisted knee extension; NJF technique, neuromuscular joint facilitation.

### 3.6 Stress-angle relationships during flexion-medial rotation (AKF, DKF, NJF)

Angle-dependent stress patterns are shown in Figure 2. For the lateral meniscus, AKF stress increased sharply from 0° to 30°, peaked at 60°, and decreased thereafter. DKF and NJF both peaked at 30° and declined progressively to 90°. Medial meniscus stress in AKF increased from 0° to 60° before decreasing sharply at 90°, whereas DKF and NJF peaked at 60° and declined to 90°. LCL stress in AKF peaked at 30° and decreased to 90°, while DKF and NJF increased steadily from 0° to 90°. MCL stress decreased from 0° to 60° in all interventions, followed by a slight increase. ACL stress in AKF and DKF decreased progressively from 0° to 90°, whereas NJF showed a decrease from 0° to 30°, an increase to 60°, and a subsequent decline. PCL stress in AKF and DKF decreased from 0° to 60° and increased thereafter, while NJF peaked at 0°, dipped at 30°, and rose again at 90°.

**Figure 2:**
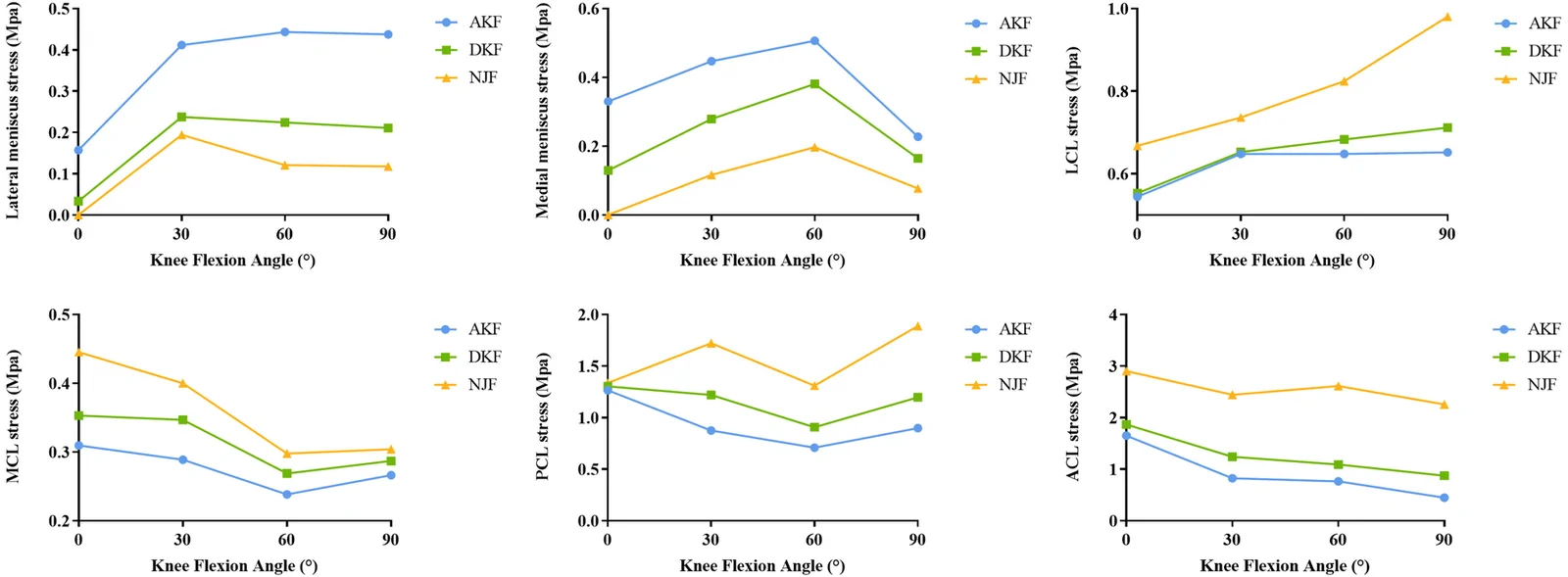
Knee flexion-tibial internal rotation movement pattern. AKF, active knee flexion; DKF, distally single-resisted knee flexion; NJF technique, neuromuscular joint facilitation; LCL, lateral collateral ligament; MCL, medial collateral ligament; ACL, anterior cruciate ligament; PCL, posterior cruciate ligament.

### 3.7 Stress-angle relationships during extension-lateral rotation (AKE, DKE, NJF)

Figure 3 illustrates stress patterns during extension-lateral rotation. Lateral meniscus stress in AKE increased from 0° to 60° before decreasing sharply at 90°, whereas DKE peaked at 30° and declined thereafter. NJF peaked at 60° and decreased to 90°. Medial meniscus stress in AKE increased steadily from 0° to 90°, while DKE and NJF peaked at 30° and declined to 90°. LCL stress in AKE increased progressively across all angles; DKE peaked at 30° and declined thereafter; NJF decreased sharply from 0° to 30° and then increased to 90°. MCL stress in all interventions decreased from 0° to 60° and increased thereafter. ACL stress in AKE and DKE decreased steadily from 0° to 90°, whereas NJF decreased sharply from 0° to 30° and increased to 90°. PCL stress in AKE and DKE increased from 0° to 30° and then declined, while NJF decreased sharply from 0° to 30° and increased to 90°.

**Figure 3:**
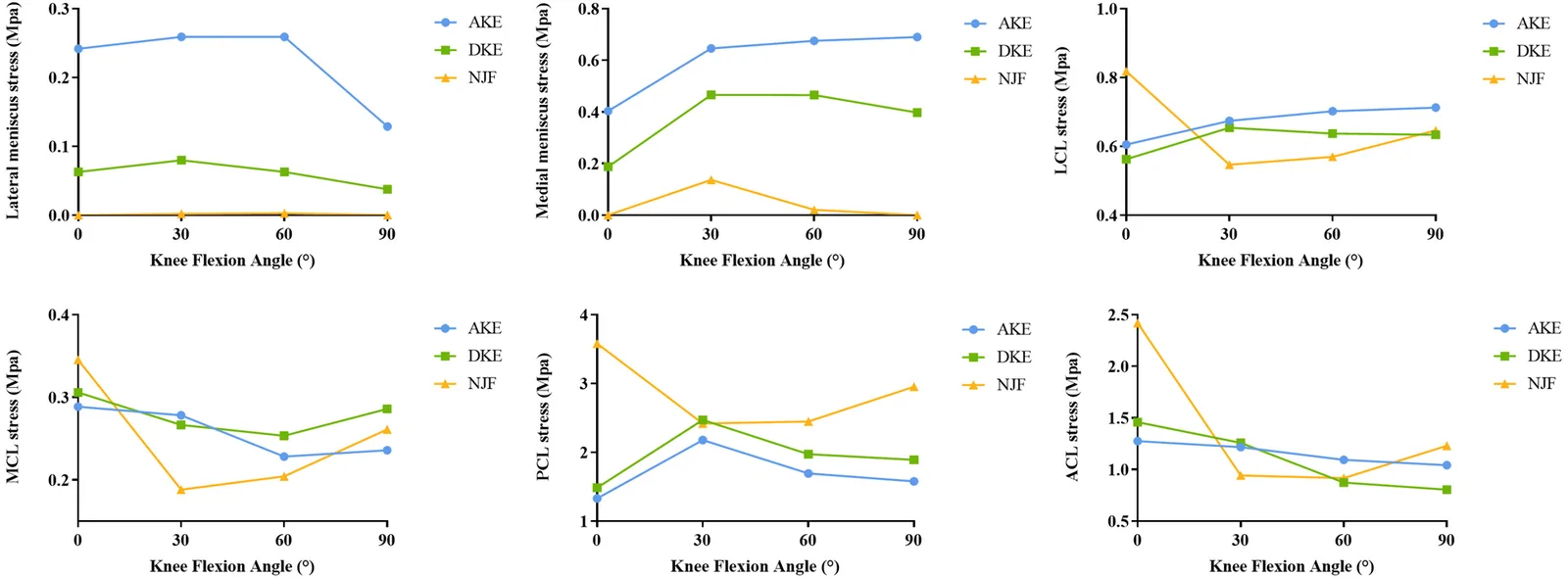
Knee extension-tibial external rotation movement pattern. AKE, active knee extension; DKE, distally single-resisted knee extension;NJF technique, neuromuscular joint facilitation; LCL, lateral collateral ligament; MCL, medial collateral ligament; ACL, anterior cruciate ligament; PCL, posterior cruciate ligament.

Figures 4–9 provide visualizations of stress distributions for the lateral meniscus, medial meniscus, LCL, MCL, PCL, and ACL within the NJF knee joint model.

**Figure 4:**
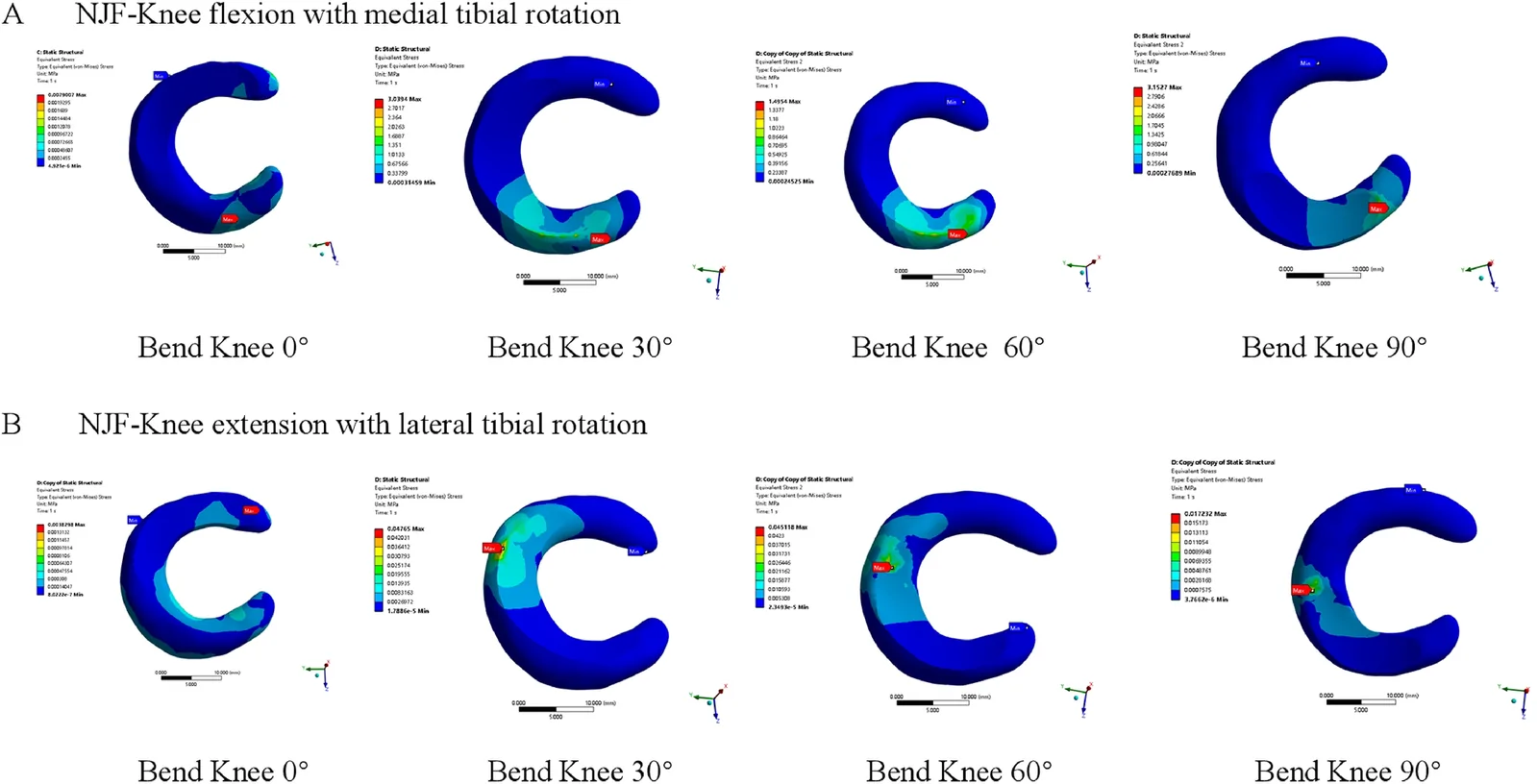
Stress variation of the lateral meniscus in the NJF knee joint model. NJF technique, neuromuscular joint facilitation. (**A**) NJF-knee flexion with medial tibial rotation at 0°, 30°, 60°, and 90° of knee bend. (**B**) NJF-knee extension with lateral tibial rotation at the same knee angles. The color map ranges from blue (minimum) to red (maximum). Red arrows and “Peak” labels indicate regions of maximum von-Mises stress, with the corresponding peak stress values (MPa) displayed.

**Figure 5:**
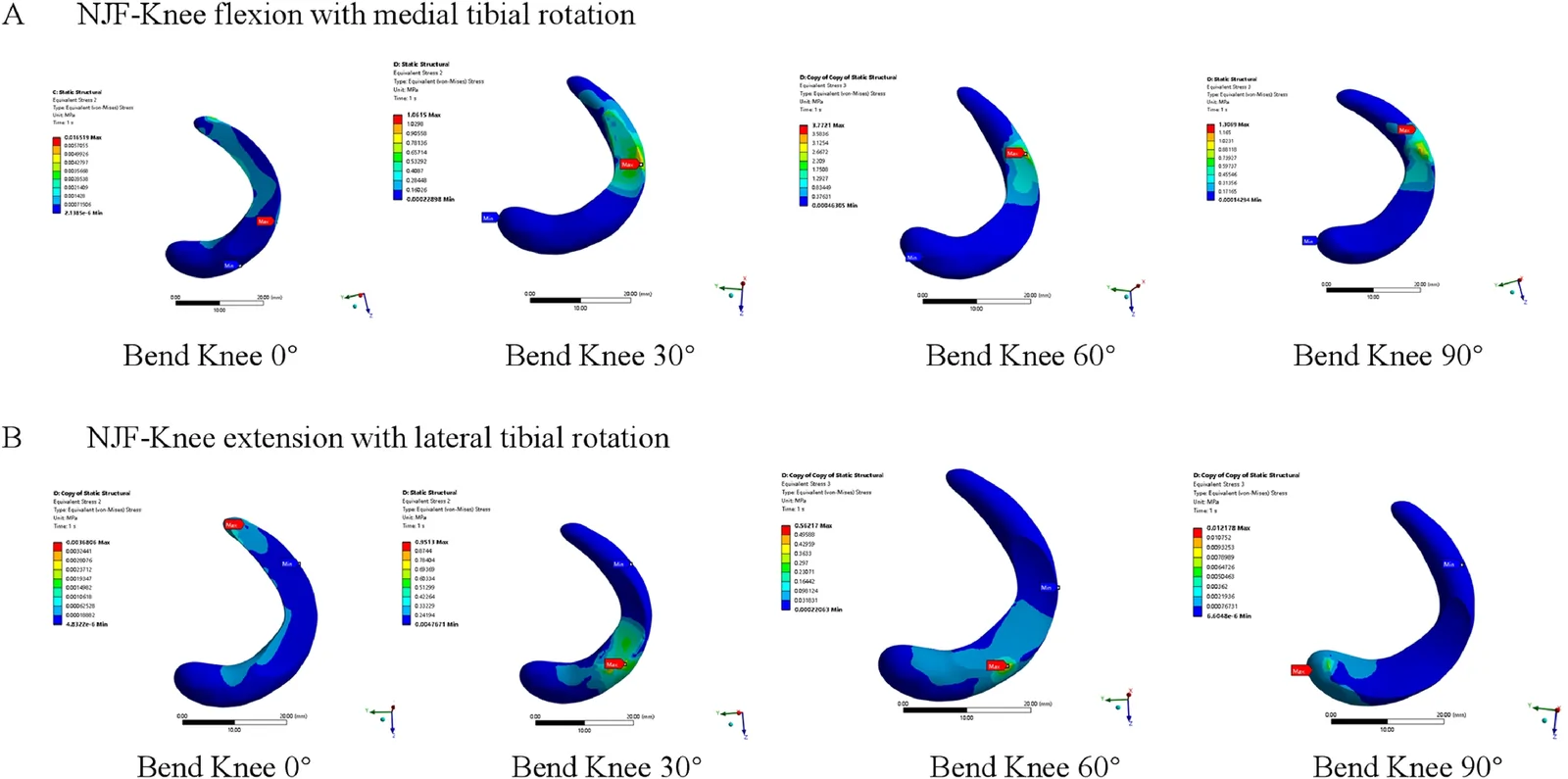
Stress variation of the medial meniscus in the NJF knee joint model. NJF technique, neuromuscular joint facilitation. (**A**) NJF-knee flexion with medial tibial rotation at 0°, 30°, 60°, and 90° of knee bend. (**B**) NJF-knee extension with lateral tibial rotation at the same knee angles. The color map ranges from blue (minimum) to red (maximum). Red arrows and “Peak” labels indicate regions of maximum von-Mises stress, with the corresponding peak stress values (MPa) displayed.

**Figure 6:**
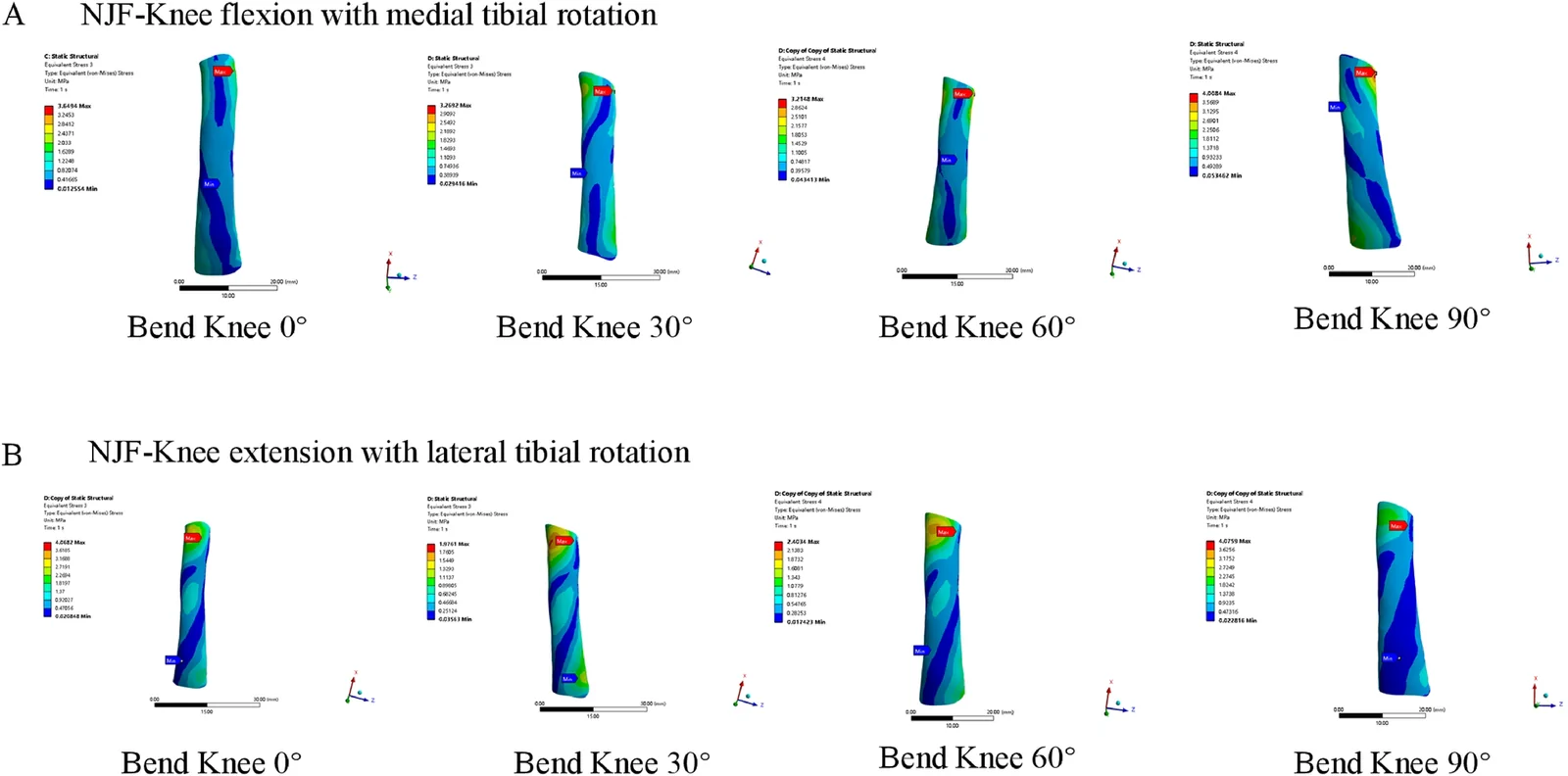
Stress variation of the LCL in the NJF knee joint model. NJF technique, neuromuscular joint facilitation; (**A**) NJF-knee flexion with medial tibial rotation at 0°, 30°, 60°, and 90° of knee bend. (**B**) NJF-knee extension with lateral tibial rotation at the same knee angles. LCL, lateral collateral ligament. The color map ranges from blue (minimum) to red (maximum). Red arrows and “Peak” labels indicate regions of maximum von-Mises stress, with the corresponding peak stress values (MPa) displayed.

**Figure 7:**
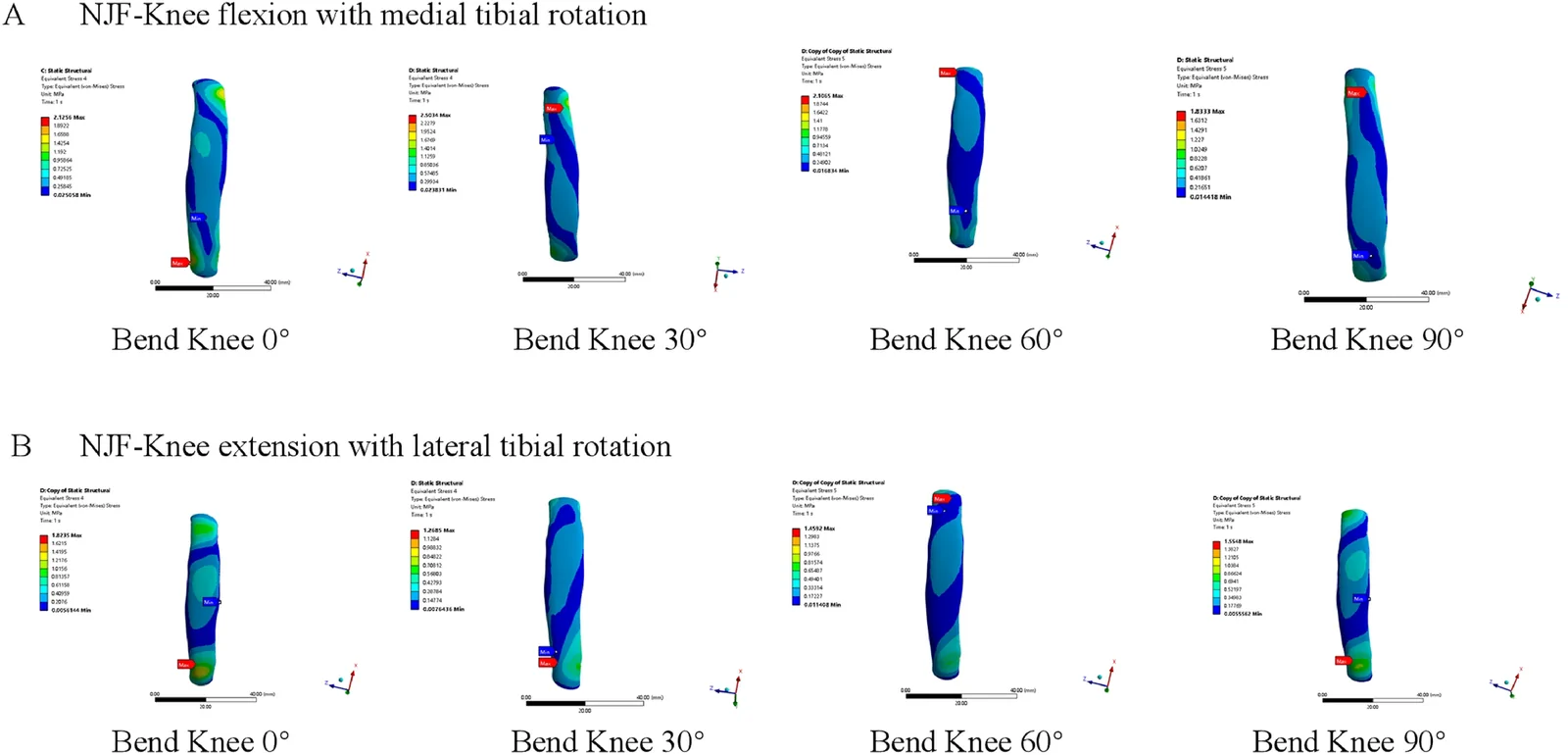
Stress variation of the MCL in the NJF knee joint model. NJF technique, neuromuscular joint facilitation; (**A**) NJF-knee flexion with medial tibial rotation at 0°, 30°, 60°, and 90° of knee bend. (**B**) NJF-knee extension with lateral tibial rotation at the same knee angles. MCL, medial collateral ligament. The color map ranges from blue (minimum) to red (maximum). Red arrows and “Peak” labels indicate regions of maximum von-Mises stress, with the corresponding peak stress values (MPa) displayed.

**Figure 8:**
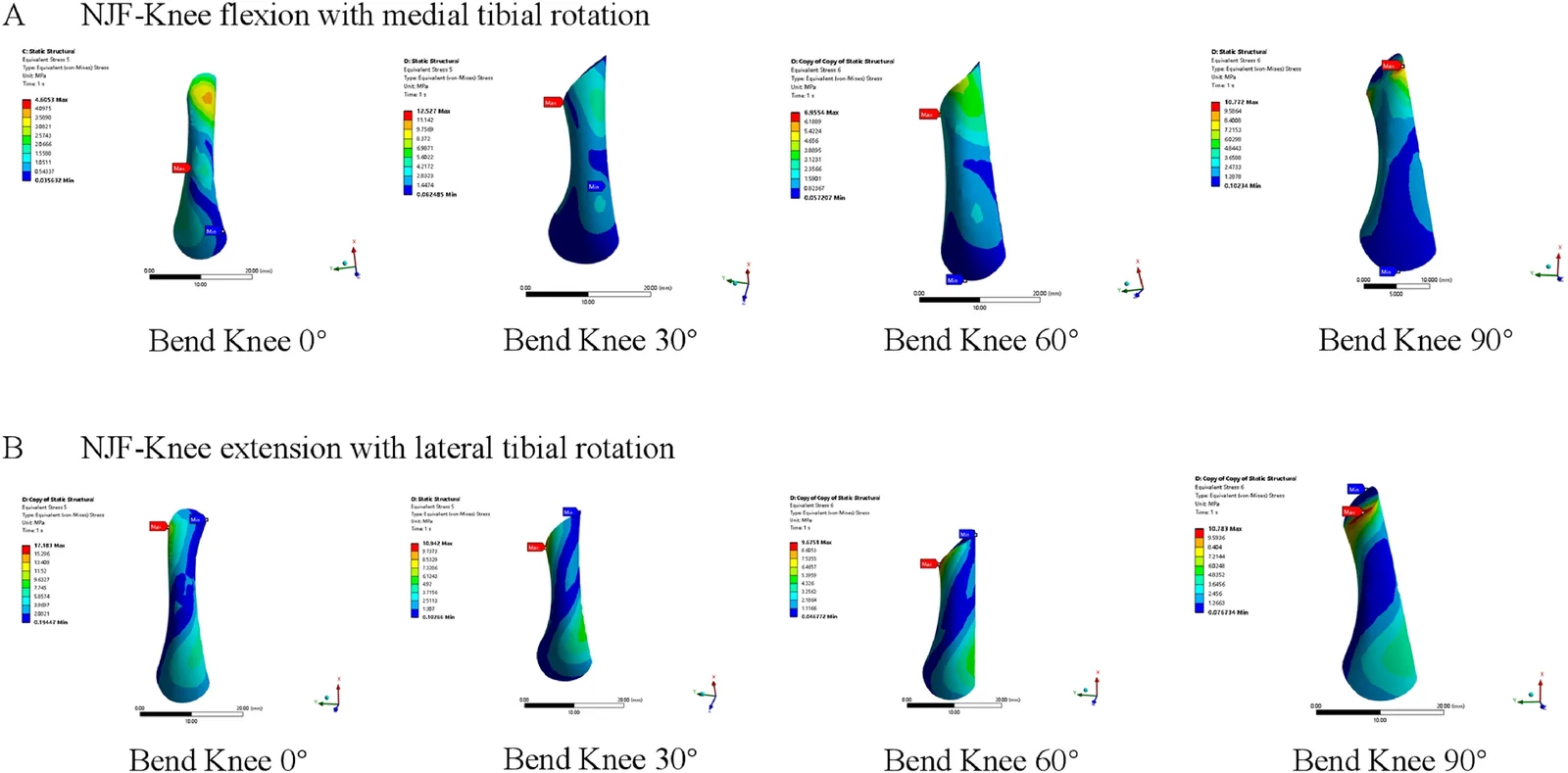
Stress variation of the PCL in the NJF knee joint model. NJF technique, neuromuscular joint facilitation; (**A**) NJF-knee flexion with medial tibial rotation at 0°, 30°, 60°, and 90° of knee bend. (**B**) NJF-knee extension with lateral tibial rotation at the same knee angles. PCL, posterior cruciate ligament. The color map ranges from blue (minimum) to red (maximum). Red arrows and “Peak” labels indicate regions of maximum von-Mises stress, with the corresponding peak stress values (MPa) displayed.

**Figure 9:**
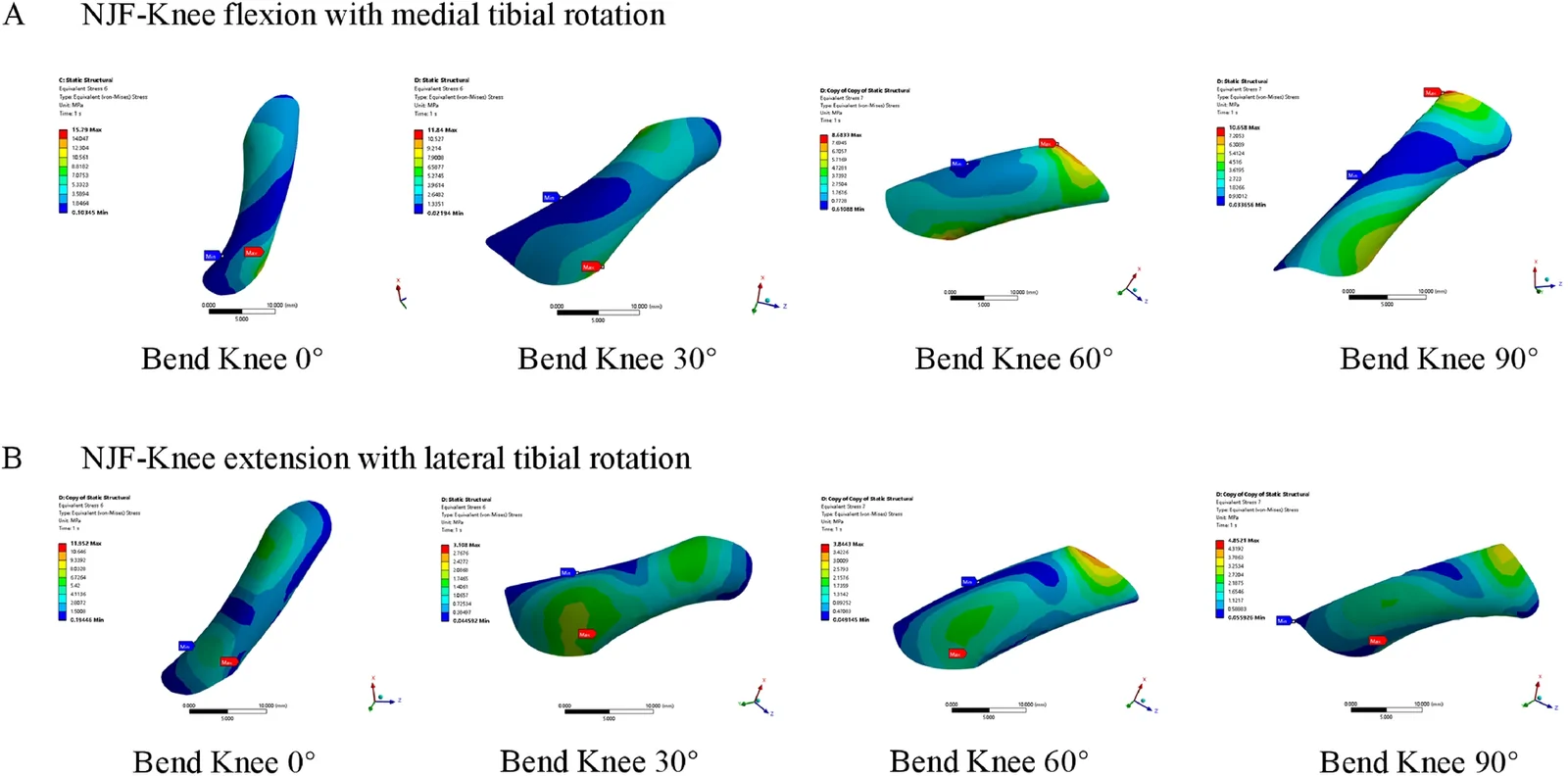
Stress variation of the ACL in the NJF knee joint model. NJF technique, neuromuscular joint facilitation; (**A**) NJF-knee flexion with medial tibial rotation at 0°, 30°, 60°, and 90° of knee bend. (**B**) NJF-knee extension with lateral tibial rotation at the same knee angles. ACL, anterior cruciate ligament. The color map ranges from blue (minimum) to red (maximum). Red arrows and “Peak” labels indicate regions of maximum von-Mises stress, with the corresponding peak stress values (MPa) displayed.

## 4 Discussion

Using finite element analysis combined with a wireless inertial motion capture system and a handheld dynamometer, this study systematically investigated the mechanisms by which NJF knee extension-lateral tibial rotation and flexion-medial tibial rotation patterns affect the stress distribution on the meniscus, ligaments, and articular surfaces in patients with KOA. Kinematic parameters and resistance were quantified, and the reliability was verified for three intervention forms: active movement (AKE/AKF), single-resistance (DKE/DKF), and NJF.In this study, “load redistribution” refers to the simultaneous reduction of meniscal stress and moderate increase of ligament mechanical stimulation induced by NJF.

Unlike previous NJF studies that primarily focused on clinical and functional outcomes (e.g., pain relief, muscle strength improvement), the present computational biomechanical approach reveals distinctive mechanical features of NJF that differentiate it from conventional resistance training. We first establish a unified mechanical framework, then elaborate on how this framework explains tissue-specific stress changes in the menisci and ligaments, and finally place these computational findings in the context of prior literature while clearly delineating the boundaries of our study and the scope of clinical inference.

### 4.1 Interpretation of meniscal stress findings

Finite element models have been shown to provide relatively accurate simulations of biomechanical problems and play an important role in noninvasively understanding knee biomechanics (18). Previous studies have shown that 3D models of KOA based on finite element analysis allow noninvasive visualization of biomechanical changes. These changes can be observed at different knee angles (11, 14). Our results indicated that in the flexion-medial tibial rotation mode, both the lateral and medial menisci showed the highest stress during non-resisted movement and the lowest stress during NJF at 0°, 30°, 60°, and 90°. This subject-specific simulation indicated that NJF substantially reduced meniscal stress in this particular model during knee movement, suggesting a potential protective effect against meniscal degeneration.

During the NJF, resistance was applied to both the distal and proximal tibia at a 30° angle to the tibial long axis in the direction opposite to the movement. The axial traction component, which acts along the tibial long axis to counteract the compressive joint reaction force, thereby increasing joint space and reducing intra-articular pressure. The transverse rotational component, which generates an effective torque to drive tibial internal rotation (in the flexion pattern) or external rotation (in the extension pattern), while distributing shear forces more evenly across the meniscal surfaces. The synergistic action of these two components constitutes the hallmark mechanical signature of NJF-it is not merely “unloading”or “overloading”, but rather a directional load redistribution. Axial traction relieves high-load regions within the joint (menisci and cartilage), while the rotational torque and dual resistance transmit moderate tensile stimulation to the peri-articular ligaments. Previous studies have also shown that knee traction effectively alleviates intra-articular pressure, increases joint space, and decreases contact pressure between articular surfaces, creating a favorable biomechanical environment for cartilage repair (19, 20).

### 4.2 Interpretation of ligament loading

The mechanical stimulation of ligament structures by NJF differs from that of traditional resistance training. In the flexion-medial tibial rotation mode, the LCL, MCL, ACL, and PCL exhibited the lowest stress during non-resisted movement and the highest stress during NJF at all angles. This suggests that NJF was found to increase ligament mechanical stimulation in the model while reducing meniscal load. Knee ligaments are not only biomechanical constraints but are also intimately related to proprioceptive function, acting as tension receptors with important neurosensory functions (21, 22). Mechanoreceptors within ligaments transform deformations into neural signals, providing the central nervous system with a sense of joint position and movement (15, 23). Therefore, NJF may potentially enhance afferent proprioceptive input, thereby facilitating coordinated peri-articular muscle activation and improving dynamic joint stability. However, it must be clearly emphasised that this hypothesis—involving proprioceptive and neuromuscular control pathways—was not directly measured in the present study, and its validation requires future investigations incorporating electrophysiological and functional assessments. Previous studies have also shown that NJF knee patterns relieve knee pain, improve muscle strength, gait, and walking speed (8).

### 4.3 Comparison with previous literature

The wireless inertial motion capture system demonstrated good consistency in measuring the knee rotation angle. ICC analysis showed reliability >0.75 for all three interventions, indicating that the system is an effective tool for collecting kinematic parameters during NJF training in patients with KOA. Koo and Li (24)suggested that an ICC >0.75 indicates good consistency, and our results further validate the clinical applicability of this measurement tool. Quantifying the kinematic and mechanical parameters of manual techniques aids in the standardization of rehabilitation techniques and provides a basis for subsequent simulation of manual loading.

The NJF movement patterns showed differential regulatory effects at different flexion angles. The flexion-medial tibial rotation pattern markedly lower meniscal stress at all angles while providing relatively balanced ligament loading in the model. Whether this translates to delayed meniscal degeneration in early-stage KOA requires further clinical investigation. In the extension-lateral tibial rotation mode, lateral and medial meniscus stress were consistently highest during non-resistance and lowest during NJF at all angles, consistent with the flexion mode findings and further validating the advantage of NJF in reducing meniscal stress. However, the ligament stress responses in the extension mode showed a clear angle dependence and structural specificity. Under NJF, ligament stress regulation was not unidirectional (increase or decrease) but showed complex interaction patterns with the flexion angle. Notably, between 30° and 60°, NJF reduced peak stress for most ligaments, whereas at 0° near full extension, it provided significant mechanical stimulation to the ligaments. This trend is consistent with those of previous biomechanical studies on knee joint movement (25, 26). These findings provides biomechanical evidence that NJF knee patterns improve knee motor functions.

### 4.4 Clinical implications

NJF redistribute load between the menisci and ligaments and reduce peak stress in high-load regions of the articular cartilage and meniscus. These computational findings demonstrate a biomechanical profile of NJF that differs from that of traditional resistance training in this subject-specific model. However, finite element analysis investigates mechanical behavior under simulated conditions; it does not directly measure clinical outcomes. Therefore, while these findings provide a theoretical basis for future investigations, they should not be interpreted as evidence of clinical superiority. Prospective clinical trials are needed to determine whether the biomechanical differences observed in this model translate into meaningful improvements in patient-reported outcomes, physical function, or disease progression.

It is important to emphasize that the physiological mechanisms discussed above-including proprioceptive enhancement, mechanoreceptor activation, and neuromuscular control-were not directly measured in this study. The interpretations offered are hypotheses generated from the observed biomechanical patterns and should be treated as such. Future mechanistic investigations incorporating electrophysiological, proprioceptive, and functional assessments are needed to confirm these potential mechanisms.

### 4.5 Methodological considerations and limitations

However, this study has some limitations. First, the static/quasi-static analysis did not capture the highly dynamic knee mechanics during rehabilitation, including time-dependent loading, muscle activation, and viscoelastic tissue behavior, thus limiting physiological realism. Second, no long-term clinical intervention studies have been conducted. Therefore, the clinical effects of NJF training require further validation.

Third, the finite element model was reconstructed from CT and MRI data of a single KOA subject. Although kinematic and mechanical parameters were collected from six patients, all stress analyses ultimately reflect the specific anatomy, cartilage thickness, ligament insertion, and material properties of this individual knee, not the entire cohort. FE outcomes are highly sensitive to subject-specific morphology. Even modest differences in lower-limb alignment, cartilage degeneration, meniscal morphology, or ligament laxity may substantially alter intra-articular stress distributions (11, 18). Therefore, inter-individual anatomical variability cannot be accounted for. These findings should not be generalised to all KOA patients without validation using additional models from different individuals.

Fourth, while the motion capture system showed acceptable relative reliability (ICC >0.75), the absolute measurement error (SEM: 1.54°–3.32°; MDC: 4.27°–9.20°) should be considered, when interpreting the kinematic inputs to the finite element model. These measurement errors may affect the absolute stress values. However, because the same measurement system was used consistently across all three intervention conditions, the relative comparisons between conditions remain valid. Bland-Altman analysis was not performed due to the limited number of participants (*n* = 6)which precludes stable estimates.

Fifth, soft tissues were modelled as linear elastic, isotropic, and homogeneous materials, which simplifies their known viscoelastic and anisotropic behaviour. Sixth, the model did not incorporate active muscle forces, which modulate joint loading *in vivo*. Seventh, tissue properties in osteoarthritic patients may differ from the literature values used, introducing additional uncertainty. Eighth, the simulated stresses lack external validation against *in vivo* measurements (e.g., pressure sensors or strain gauges), and absolute stress values should be interpreted as comparative indices rather than true physiological stresses. Ninth, all stress values are deterministic numerical solutions under idealised assumptions. They reflect computational precision—the internal consistency of the solver—but not biological accuracy (true *in vivo* stresses). Absolute magnitudes depend on assumed properties and should be interpreted as comparative indices for ranking interventions, not as exact tissue loads. Tenth, no sensitivity analysis was conducted for material properties, boundary conditions, loading parameters, or friction coefficients; given the inherent sensitivity of finite element predictions, future studies should evaluate their effects on model robustness and the computational results.

### 4.6 Future directions

These computational findings demonstrate that NJF redistributes load between the menisci and ligaments in this subject-specific model. This biomechanical profile differs from that of traditional resistance training and provides a theoretical foundation for future clinical investigations. However, the clinical relevance of these biomechanical observations remains to be established through prospective studies incorporating functional and physiological outcome measures. Future research should include multicenter, large-sample, randomized controlled trials to verify the generalizability and clinical translational value of these findings. Future studies should also integrate subject-specific musculoskeletal modelling to incorporate active muscle forces (quadriceps, hamstrings, gastrocnemius) and co-contraction patterns, enabling a more complete simulation of *in vivo* joint loading during NJF performance.

## 5 Conclusion

This subject-specific finite element model was driven by experimentally measured kinematic and mechanical parameters. Under NJF knee extension-lateral rotation and flexion-medial rotation patterns, meniscal peak stress consistently decreased while ligamentous stimulation moderately increased across multiple flexion angles. This load-redistribution pattern differs from that observed under non-resisted and single-resisted conditions. The stress values reported are deterministic numerical solutions under idealised assumptions and should be interpreted as comparative indices, not as exact *in vivo* tissue stresses. These computational findings suggest a potentially favourable mechanical environment for the meniscus and ligaments in early-stage KOA. However, clinical outcomes—including pain relief, gait improvement, and disease progression-were not measured and remain speculative. Therefore, these results should not be interpreted as evidence of biomechanical advantages. These findings should be interpreted as representative of the investigated subject-specific computational model, not as universally applicable to all individuals with KOA. They provide a biomechanical rationale for future investigations. Prospective randomised controlled trials with patient-reported outcomes and functional assessments are needed to confirm clinical translation. Further computational studies across different KOA severities are also required to test generalisability. Future studies employing patient-specific finite element models across larger cohorts with varied anatomical presentations are needed to improve external validity.

## Data Availability

The raw data supporting the conclusions of this article will be made available by the authors, without undue reservation.
